# Effectiveness of a community-based peer support service among persons suffering severe mental illness in China

**DOI:** 10.7717/peerj.14091

**Published:** 2022-10-10

**Authors:** Yunge Fan, Ning Ma, Aili Ouyang, Wufang Zhang, Manxi He, Yong Chen, Jin Liu, Zhongxiang Li, Junlan Yang, Liang Ma, Eric D. Caine

**Affiliations:** 1 Peking University Sixth Hospital; Peking University Institute of Mental Health; Key Laboratory of Mental Health, Ministry of Health (Peking University); National Clinical Research Center for Mental Disorders (Peking University Sixth Hospital), Beijing, China; 2 The Fourth People’s Hospital of Chengdu, Chengdu, Sichuan, China; 3 Pengzhou Mental Health Center, Chengdu, Sichuan, China; 4 Department of Psychiatry, Chengdu Dekang Hospital, Chengdu, Sichuan, China; 5 Third hospital of Chaoyang district, Beijing Chaoyang District Mental Disease Prevention and Control Center, Beijing, China; 6 Department of Psychiatry, University of Rochester Medical Center, New York, Rochester, NY, USA

**Keywords:** Peer support service, Community, Severe mental illness, Effectiveness, China

## Abstract

**Background:**

Community-based peer support service is widely and effectively deployed for persons suffering severe mental illness (SMI) in countries with well-developed outpatient mental health systems. The objective of this study is to evaluate the effectiveness of a 1-year peer service project among persons with SMI implemented in China.

**Methods:**

A total of 101 consumers (service recipients) and 66 family caregivers were recruited at baseline from communities located in Beijing and Chengdu. Severity of psychiatric symptoms, personal and social functioning, self-esteem, life satisfaction, and medication adherence were evaluated among consumers. Self-esteem, life satisfaction, anxiety, and depressive symptoms were assessed among family caregivers. Participants were reevaluated at 1 year with the same measures. Changes in outcomes from baseline to 1-year follow-up were examined using paired sample *t* tests or Stuart-Maxwell tests.

**Results:**

Consumers’ psychiatric symptoms were decreased at 1 year (*p* < 0.001). Their personal and social functioning (*p* = 0.003) and life satisfaction (*p* < 0.001) were increased. There were no improvements in self-esteem (*p* = 0.108) and medication adherence (*ps* ≥ 0.827) among consumers. For caregivers, no increases were presented in outcomes at the 1-year assessment (*ps* ≥ 0.164).

**Conclusions:**

The findings suggest that peer support services could be sustainably implemented across China, with positive impacts on the psychiatric symptoms, social functioning, and life satisfaction of participants suffering SMI.

## Introduction

Community-based peer support services are widely deployed for persons suffering severe mental illness (SMI) in many Westernized countries (Miyamoto & Sono, 2012; Repper & Carter, 2011; Trainor et al., 1997). It is based on the premise that individuals who have recovered from mental illness (*e.g.*, schizophrenia and bipolar disorder) can be positive role models and offer support, encouragement, and guidance to others facing similar situations (Davidson et al., 2012; Davidson et al., 2006; Lawn, Smith & Hunter, 2008). Involvement of peers (*i.e.*, peer service providers) in care is new to most nations that now are developing outpatient programming. Previous research has suggested that the implementation of peer support services should be consistent with local customs, values, and resource availability (Solomon, 2004).

The effects of community-based peer support services for SMI are multi-faceted. Our prior studies have documented that both the direct recipients of peer support services (hereafter, “consumers”) and their family caregivers may benefit from the implementation of these services (Fan et al., 2019). For consumers, existing research has demonstrated that peer-delivered services can improve their self-esteem and self-efficacy, feelings of understanding, sense of respect and trust, and life satisfaction (Fukui et al., 2010; Mahlke et al., 2017). One study that evaluated the effects of a 12-week peer-run service for people with major psychiatric problems found improvement in self-efficacy even three months after the service (Van Gestel-Timmermans et al., 2012). However, previous studies have shown inconsistent findings concerning psychiatric symptoms and social functioning. One study implemented a 12-week peer support service for 47 outpatients with severe and persistent mental illness, including schizophrenia, bipolar disorder, schizoaffective disorder, and major depressive disorder. The core activities of each service session consist of group discussion, reading recovery workbook, small group activities, personal reflection, and writing in the workbook (Fukui et al., 2010). The results of pre-post service evaluations revealed alleviation of psychiatric symptoms, such as hostility, paranoid ideation, psychoticism, depression, anxiety, and conceptual disorganization. An 8-week peer intervention recruited adults with severe and persistent mental illness (*e.g.*, schizophrenia, bipolar disorder, and depressive disorder) from outpatient community mental health settings (Cook et al., 2011). The results also showed a reduction in psychiatric symptoms over time. Yet another inpatient randomized trial found that peer support intervention improved recipients’ social and personal functioning but not their psychiatric symptoms (Rogers et al., 2016). A 6-month one-to-one peer support service found no differences between the intervention and control groups for rehospitalization and social functioning (Mahlke et al., 2017).

Family caregivers of persons suffering SMI often report great burden and emotional distress (Barker et al., 2012; Magliano et al., 1998). An evaluation of a 3-month community-based peer support program indicated that the service caregivers feel more confident about the recovery process of their mentally ill family members (Lawn, Smith & Hunter, 2008). Although there were no sessions delivered to the caregivers directly, seeing a peer worker who was similar to the ill member has given the caregivers hope for the recovery of their own ill family members. In addition, as the ill family members became more and more independent after participating in the peer support service, caregivers themselves are likely to experience improved mood, reduced caring burden, and more confidence in their family member’s recovery progress (Fan et al., 2019). However, few studies have systematically assessed the effect of peer-delivered services on family caregivers using quantitative measures.

There have been several notable limitations in prior studies. The majority have come from Western countries with well-developed community services. To date, the impact of peer services delivered in Chinese communities is still not clear. In addition, most studies provided their follow-up evaluations at 3 to 6 months (Cook et al., 2011; Fukui et al., 2010; Lawn, Smith & Hunter, 2008; Van Gestel-Timmermans et al., 2012). Longer-term influences of peer support services have received little attention. Finally, families of persons suffering SMI are key stakeholders, but few studies have examined the effect on their well-being. Prior studies have consistently demonstrated that family caregivers of persons with SMI experience significant stress and have a high level of burden (Chan, 2011; Saunders, 2003). Conversely, family caregivers’ well-being, preparation, and knowledge also influence the outcomes of their ill family members (Meleis et al., 2000). Therefore, exploring the influence of peer support service on family caregivers’ well-being is important to comprehensively capture the ways through which the service takes effect.

In hopes of filling the gaps in prior research, this study is aimed at investigating the effectiveness of a 1-year community-based peer support service in China among persons suffering SMI, who were involved in the community follow-up service of the Basic Public Health Services Project in China, and their family caregivers. First, it was hypothesized that consumers, who have participated in the service, would experience a reduction in psychiatric symptoms and improvements in personal and social functioning, self-esteem, life satisfaction, and medication adherence. Second, it was hypothesized that there would be increases in self-esteem and life satisfaction, and decreases in anxiety and depression among family caregivers after their ill family members participated in the service for 1 year.

## Materials & Methods

### Participants

A total of 101 consumers (service recipients) and 66 family caregivers were recruited at baseline from communities located in Beijing and Chengdu. Peer support services were developed in two communities in Beijing in August 2015. Thirty consumers (*i.e.*, peer service recipients) were recruited from the two communities. To enlarge the sample size and verify the outcomes of peer support services, the program was developed further in two communities of Chengdu in November 2019. Seventy-one consumers were enrolled from the later launched communities. In total, the program was applied in four communities in the two cities (Beijing and Chengdu). The detailed numbers of participants assessed at each time point in each site were shown in Table 1.

**Table 1:** The number of participants was assessed at each time point in each site.

City	Assessment time	Consumers (n)	Family caregivers (n)
Beijing (two communities)	Baseline assessment (Aug to Dec 2015)	30	26
	1-year follow-up assessment (Aug to Dec 2016)	21	14
Chengdu (two communities)	Baseline assessment (Nov to Dec 2019)	71	40
	1-year follow-up assessment (Dec 2020)	57	27
In total (four communities)	Baseline assessment	101	66
	1-year follow-up assessment	78	41

The recruitment of participants was based on posters and recommendations from community doctors. The inclusion criteria of consumers included: diagnosed with schizophrenia or bipolar disorder according to the medical record; age between 18 and 60 years old; mental illness conditions stable for at least 3 months; no drug or alcohol abuse. The follow-up assessment was conducted 1 year after the initial evaluation.

Among the 101 consumers recruited at baseline, 78 (77.2%) were assessed at the 1-year follow-up. In addition, caregivers of participants were also assessed if they were available; 66 and 41 caregivers were evaluated at baseline and 1-year follow-up, respectively.

The project proposal was reviewed and approved by the Ethics Committee of Peking University Sixth Hospital (approval reference number: 2012-3) prior to its initiation. All consumers provided written informed consent, and their caregivers provided written or verbal informed consent.

### Procedures

At the baseline assessment, each participant was interviewed face-to-face by a psychiatrist in the research team to evaluate his/her eligibility and to collect socio-demographic information and relevant medical history. Severity of psychiatric symptoms, personal and social functioning, self-esteem, and life satisfaction were also evaluated. Consumers who participated in the service were reevaluated at 1 year after the baseline evaluation with the same measures. All the evaluations were self-report questionnaires filled in as oral-reported while assisted by the psychiatrists and research assistants in the research team. Peer support services were ongoing at the follow-up evaluation. All participants also received usual care while they were receiving peer support interventions. Moreover, the available caregivers of participants were interviewed individually and face-to-face by trained research assistants to assess their self-esteem, life satisfaction, anxiety, and depression at baseline and the follow-up.

### Peer support intervention

A total of 87 sessions were delivered in the four communities (45 sessions in the two communities of Beijing and 42 sessions in the two communities of Chengdu) during 1 year. The peer support services were conducted in groups. One session refers to that peer service providers met with consumers once and conduct activities or support service in groups. Each session was provided once every two weeks. The average attendance for each consumer was 59%. Each session had a specific topic and the topics can be summarized into eight categories: daily life skills, social skills, knowledge of mental disorders, entertainment, fine motor skill practice, personal perceptions, healthy lifestyle support, and emotional support. Among the 87 sessions, seven (8%) sessions were classified as daily life skills, eight (9%) were social skills, nine (10%) were knowledge of mental disorders, 14 (16%) were entertainment, 15 (17%) were fine motor skill practice, two (2%) were personal perceptions, 12 (14%) were healthy lifestyle support, and 20 (23%) were emotional support. The format of each service session varied depending on the topics, including group discussion, role play, personal sharing, lecture, and outdoor exercise. The duration of each service session ranged from 40 min to 120 min depending on the activity topics and format for the session. Each session typically involved at least two peer service providers, with one leading the session and the other one assisting with service delivery, record keeping, and documenting consumers’ feedback. There were usually three parts for each session: brief warm-up activities (*e.g.*, reading peer support rules, icebreaking games, or setting-up exercises), main activities related to each topic, and ending activity (*e.g.*, an overview of the session, reciting peer support poem, or singing a song). The majority of service sessions were held in community rehabilitation centers or community health care centers, except for outdoor activities. A detailed description of the peer support intervention was published in a prior article by the research team (Fan et al., 2018).

### Measures

#### Consumer

##### Psychiatric Symptom.

The Positive and Negative Syndrome Scale (PANSS) was used to assess the psychiatric symptoms of consumers (Bowie et al., 2010; Kay, Fiszbein & Opfer, 1987). It is a well-developed scale with good reliability and validity. The Cronbach’s *α* coefficients were .73 to .83. The Chinese version of PANSS has previously been published and demonstrated good reliability (Cronbach’s *α* = .74 to .90) and validity (Tianmei et al., 2004). It contains 30 items with seven measuring positive symptoms, seven measuring negative symptoms, and 16 items measuring general aspects of psychopathology. Each item score ranges from 1 to 7 with 1 representing the absence of symptom and 7 representing extremely serious symptom. Sum scores of each subscale and the whole scale were calculated with higher scores indicating more severe symptoms.

##### Personal and social functioning.

The Personal and Social Performance Scale (PSP) was used to measure the personal and social functioning of consumers (Morosini et al., 2000; Tianmei et al., 2011). It was developed based on the social functioning component of the DSM-IV Social and Occupational Functioning Assessment Scale (SOFAS). The Chinese version of PSP has previously been published and demonstrated good reliability and validity with a Cronbach’s *α* being .84 (Tianmei et al., 2011). The scale is a 100-point single-item rating scale with higher scores representing better personal and social functioning. The ratings are based on four main areas: (a) socially useful activities, including work and study; (b) personal and social relationships; (c) self-care; and (d) disturbing and aggressive behaviors.

##### Self-esteem.

The Rosenberg’s Self-Esteem Scale (SES) was used to evaluate consumers’ self-esteem (Blascovich et al., 1991; Rosenberg, 2015). It is a widespread self-report scale with 10 items. Each item scores from 1 (strongly disagree) to 4 (strongly agree). A sum score of the scale was calculated and used in the analysis, with higher scores indicating better self-esteem. The Chinese version of SES has previously been published and demonstrated good reliability (Cronbach’s *α* = .80 to .89) and validity (Chen, Bi & Han, 2015).

##### Life satisfaction.

The Life Satisfaction Index A (LSIA) was used to measure the perceived quality of life (Neugarten, Havighurst & Tobin, 1961). It is a well-developed self-report scale including 20 items. Each item is scored 1 or 0 to indicate that the participant agrees or disagrees, respectively, with the statement. A sum score of all items was used in the analysis, with higher values representing higher perceived life satisfaction. The Chinese version of LSIA has previously been published and demonstrated good reliability (Cronbach’s *α* = .73 to .75) and validity (Jiang et al., 2018).

##### Socio-demographic and medical characteristics.

A self-designed questionnaire was used to collect participants’ socio-demographic and medical characteristics, including age, gender, education level, marital status, employment status, whether living alone, diagnosis of mental illness, first onset age of mental illness, source of personal income, perceived financial pressure due to mental illness, and medication adherence. Medication adherence was assessed with two items. The first item is the regularity of taking medications, for which three options are included: “taking medication regularly”, “taking medication irregularly”, and “not taking medication”. The second item is the independence of taking medications, including four options: “self-reminded to take medication”, “others-reminded to take medication”, “both self-reminded and others-reminded to take medication”, and “not taking medication”.

#### Caregiver

##### Self-esteem.

The measure of self-esteem for caregivers is identical to that for consumers.

##### Life satisfaction.

The measure of life satisfaction for caregivers is identical to that for consumers.

##### Anxiety.

The Self-Rating Anxiety Scale (SAS) was used to assess the anxiety symptoms of caregivers (Zung, 1971). It is a well-validated measure to screen anxiety over the course of past week with the split-half correlations being .83 to .71 (Zung, 1971). The Chinese version of SAS has previously been published (Liu et al., 1997). It is a self-reported scale including 20 items, among which 15 are to measure somatic symptoms and 5 are affective symptoms. Caregivers rated the extent to which one experienced each anxiety symptom on a 4-point scale. An index score, calculated by multiplying the total raw score by 1.25, was used in the analysis. As a result, the total index score ranges between 25 and 100 with higher scores indicating more severe anxiety symptoms. By transforming the raw score to an index score, we can compare the results with other studies using the same measure and explore the anxiety severity of the caregivers (Zung, 1971).

##### Depression.

The Self-Rating Depression Scale (SDS) was used to assess the depressive symptoms of caregivers (Zung, 1965). It is a widespread instrument to screen affective, psychological, and somatic symptoms associated with depression over the past week. The Chinese version of SDS has previously been published with a Cronbach’s *α* coefficient being .91 and split-half reliability being .89 (Lee et al., 1994). It is a self-reported scale with 20 items. Caregivers rated the severity of each symptom that they experienced on a 4-point scale. An index score, calculated by multiplying the total raw score by 1.25, was used in this study. The index score ranges between 25 and 100 with higher scores indicating more severe depressive symptoms. Similar to SAS, transforming the raw score to index score allows us to explore the depressive severity of the caregivers (Zung, 1965).

##### Socio-demographic characteristic.

A self-designed questionnaire was used to collect caregivers’ age, gender, education level, and employment status.

### Statistical analysis

The statistical analysis was conducted with the SPSS 25.0 for Windows. First, descriptive statistics of the socio-demographic and medical characteristics of the consumers and caregivers at baseline were calculated. To compare the differences between consumers who remained in the study at the follow-up assessment and those who did not, independent sample *t* tests and Chi-square tests were conducted. Next, the changes in outcome variables of consumers and caregivers from baseline to 1-year follow-up were examined. Paired sample *t* tests were used to investigate the changes in continuous variables, and Stuart-Maxwell tests were used to examine the changes in category variables. Finally, to examine whether dropouts affected the results, missing values were replaced with the series means. Additional paired sample *t* tests were conducted with the replaced data set and the results were compared with those from the original data set.

## Results

### Participant characteristics

The descriptive statistics of consumers’ baseline socio-demographic and medical characteristics are listed in Table 2. Participants were 101 consumers with a mean age of 46.69 (SD = 10.98) years at baseline. Forty-seven (46.5%) were males. Most of them completed middle school (*n* = 29, 28.7%) or high school (*n* = 30, 29.7%), and approximately one in five attained a diploma or higher degree (*n* = 20, 19.8%). More than half of them were married or partnered (*n* = 52, 51.5%). Approximately one-tenth (*n* = 11, 10.9%) were working full-time, 6 (5.9%) were working part-time, and the majority (*n* = 84, 83.2%) were not employed. The majority were living with family members and approximately one in ten were living alone (*n* = 10, 9.9%). Most of the consumers had a diagnosis of schizophrenia (*n* = 96, 95.0%), while 5 (5.0%) were diagnosed with bipolar disorder. The average first onset age was 27.51 (SD = 10.96) years. At the 1-year follow-up evaluation, 78 (response rate 77.2%) consumers are assessed.

**Table 2:** Description of the consumers (*N* = 101) characteristics at baseline.

Variables	n (%)	Mean (SD)
Age, mean (SD)		46.69 (10.98)
Male	47 (46.5)	
Education		
<Middle school	21 (20.8)	
Middle school	29 (28.7)	
High school	30 (29.7)	
Junior College	10 (9.9)	
College or more	10 (9.9)	
Marital status		
Never married	34 (33.7)	
Married or partnered	52 (51.5)	
Divorced or separated	13 (12.9)	
Employment		
Full-time employed	11 (10.9)	
Part-time employed	6 (5.9)	
Not employed	84 (83.2)	
Living alone	10 (9.9)	
Diagnosis		
Schizophrenia	96 (95.0)	
Bipolar disorder	5 (5.0)	
First onset age, mean (SD)		27.51 (10.96)
Source of personal income		
Government subsidy	93 (92.1)	
Retirement allowance	27 (26.7)	
Perceived financial pressure due to mental illness		
No	31 (34.8)	
Low	30 (29.7)	
Median	19 (18.8)	
High	9 (8.9)	
Extremely high	2 (2.0)	

There were 23 dropouts at the 1-year follow-up. Compared to the consumers available at the 1-year evaluation, those who dropped out were more likely to be younger (41.39 vs. 48.26; *t* (99) = −2.72, *p* = 0.008). No other differences were detected in any socio-demographic and medical characteristics, psychiatric symptoms, personal and social functioning, self-esteem, and life satisfaction variables between the two groups. In addition, Little’s Missing Completely at Random (MCAR) test (Little & Rubin, 1989) was performed to analyze the missing values in all variables, and the results revealed that the data were missing at random (*χ*^2^(119) = 125.36, *p* = 0.327).

Among the 66 caregivers at baseline, 35 (53%) were males. The average age of the caregivers was 60.33 (SD = 15.59) years. Over one-fifth (*n* = 15, 22.7%) did not complete middle school, 25 (37.9%) finished middle school, 15 (22.7%) completed high school, and 11 (16.7%) attained a diploma or higher degree. Approximately one-fourth (*n* = 18, 27.3%) were working full-time, six (9.1%) were working part-time, and the majority (*n* = 42, 63.6%) were not employed. From the perspective of caregivers, 23 (34.8%) perceived no financial pressure due to their ill family member’s mental illness, 21 (31.8%) reported low financial pressure, 12 (18.2%) perceived median pressure, nine (13.6%) and one (1.5%) reported high and extremely high pressure, respectively. At 1 year, the caregivers of those consumers who completed the follow-up evaluation were approached, while the caregivers of those consumers who dropped out were not contacted. Forty-one (response rate 62.1%) caregivers were assessed at the 1-year follow-up. The MCAR test showed that the data of caregivers were likely to missing at random (*χ*^2^(27) = 36.50, *p* = 0.105).

### Outcomes of Consumers

Results of changes in psychiatric symptoms, personal and social functioning, self-esteem, life satisfaction, and medication adherence among consumers from baseline to 1 year after participating in peer support service are shown in Table 3. For psychiatric symptoms, the total scores of PANSS decreased significantly at 1-year follow-up compared to baseline (*t* (75) = 4.89, *p* < 0.001, ${\eta }_{p}^{2}$ = 0.242). The scores of positive symptoms (*t* (75) = 2.70, *p* = 0.009, ${\eta }_{p}^{2}$ = 0.089), negative symptoms (*t* (75) = 5.29, *p* < 0.001, ${\eta }_{p}^{2}$ = 0.272), and general aspects of psychopathology (*t* (75) = 3.10, *p* = 0.003, ${\eta }_{p}^{2}$ = 0.114) were also significantly reduced at 1 year. Moreover, compared to baseline, consumers’ personal and social functioning (*t* (74) = −3.09, *p* = 0.003, ${\eta }_{p}^{2}$ = 0.114) and life satisfaction (*t* (75) = −4.11, *p* < 0.001, ${\eta }_{p}^{2}$ = 0.184) were significantly improved at 1-year follow-up, while the improvement in self-esteem was not significant (*t* (74) = −1.63, *p* = 0.108, ${\eta }_{p}^{2}$ = 0.035).

**Table 3:** Results of psychiatric symptom, personal and social functioning, self-esteem, life satisfaction, and medication adherence outcomes at baseline and 1-year follow-up among consumers.

Variables	Baseline (*n* = 101)			1 year (*n* = 78)			Test of difference^a^	Effect size (${\eta }_{p}^{2}$)
	Mean (SD)/*n* (%)	Skewness	Kurtosis	Mean (SD)/*n* (%)	Skewness	Kurtosis		
Psychiatric symptom^b^								
Positive symptom scores	10.54 (3.21)	0.60	−0.62	9.40 (2.50)	1.09	0.85	*t* (75) = 2.70, *p* = 0.009	0.089
Negative symptom scores	14.28 (6.23)	0.58	−0.60	11.49 (4.15)	0.87	−0.06	*t* (75) = 5.29, *p* < 0.001	0.272
General aspects of psychopathology scores	24.79 (5.71)	0.61	−0.08	22.46 (3.52)	0.30	−0.63	*t* (75) = 3.10, *p* = 0.003	0.114
Total scores	49.61 (12.81)	0.46	−0.75	43.35 (8.05)	0.46	−0.95	*t* (75) = 4.89, *p* < 0.001	0.242
Personal and social functioning^c^	70.68 (12.12)	−0.72	2.47	74.95 (8.57)	0.37	0.24	*t* (74) = −3.09, *p* = 0.003	0.114
Self-esteem^d^	28.77 (5.29)	1.32	5.12	29.96 (5.19)	0.39	−0.43	*t* (74) = −1.63, *p* = 0.108	0.035
Life satisfaction^e^	11.86 (4.23)	0.39	−0.43	13.69 (4.09)	−0.27	−0.81	*t* (75) = −4.11, *p* < 0.001	0.184
Medication adherence								
Regularity of taking medications							*χ*^2^ <0.01, *p* = 1.000	
Taking medication regularly	86 (85.1%)			70 (89.7%)				
Taking medication irregularly	12 (11.9%)			6 (7.7%)				
Not taking medication	2 (2.0%)			2 (2.6%)				
Independence of taking medications							*χ*^2^= −0.218, *p* = 0.827	
Self-reminded to take medication	72 (71.3%)			61 (78.2%)				
Others-reminded to take medication	22 (21.8%)			14 (17.9%)				
Both self-reminded and others-reminded	4 (4.0%)			1 (1.3%)				
Not taking medication	2 (2.0%)			2 (2.6%)				

**Notes.**

a Continuous variables were tested by paired sample *t* test, and category variables were tested by Stuart-Maxwell test.

b Measured by the Positive and Negative Syndrome Scale (PANSS), with the score range being between 7 and 49 at positive symptom subscale and negative symptom subscale, the score range being 16 and 112 at general aspects of psychopathology subscale, and total score range being 30 and 210 at the whole scale. Higher scores indicate more severe psychiatric symptom.

c Measured by the Personal and Social Performance Scale (PSP), with the score range being 1 and 100 and higher scores indicating better personal and social functioning.

d Measured by the Rosenberg’s Self-Esteem Scale (SES), with the score range being 10 and 40 and higher scores indicating better self-esteem.

e Measured by the Life Satisfaction Index A (LSIA), with the score range being 0 and 20 and higher scores indicating better life satisfaction.

For medication adherence, including regularity of taking medications and independence of taking medications, the improvements over the course of the year were not statistically significant: 86 (85.1%) consumers took their medications regularly at the outset and 70 (89.7%) after 1 year. Similarly, 72 (71.3%) took them without others’ intervention at the beginning of the study; 61 (78.2%) did so at the year’s end.

### Outcomes of Caregivers

For caregivers (see Table 4), their family members’ participation in peer support services did not significantly improve their self-esteem (*t* (40) = −0.36, *p* = 0.719, ${\eta }_{p}^{2}$ = 0.003), life satisfaction (*t* (40) = −0.69, *p* = 0.493, ${\eta }_{p}^{2}$ = 0.012), anxiety (*t* (39) = −1.42, *p* = 0.164, ${\eta }_{p}^{2}$ = 0.049), or depression (*t* (39) = −1.05, *p* = 0.298, ${\eta }_{p}^{2}$ = 0.028).

**Table 4:** Results of self-esteem, life satisfaction, anxiety, and depression outcomes at baseline and 1-year follow-up among caregivers.

Variables	Baseline (*n* = 66)			1 year (*n* = 41)			Test of difference^a^	Effect size (${\eta }_{p}^{2}$)
	Mean (SD)	Skewness	Kurtosis	Mean (SD)	Skewness	Kurtosis		
Self-esteem^b^	31.39 (4.45)	0.17	−0.17	31.41 (4.80)	0.30	0.53	*t* (40) = −0.36, *p* = 0.719	0.003
Life satisfaction^c^	12.76 (4.84)	−0.68	−0.22	14.24 (4.06)	−0.82	0.39	*t* (40) = −0.69, *p* = 0.493	0.012
Anxiety^d^	37.19 (9.63)	1.56	4.14	38.13 (11.40)	1.28	1.66	*t* (39) = −1.42, *p* = 0.164	0.049
Depression^e^	38.09 (11.74)	1.28	1.66	38.38 (12.60)	1.36	1.50	*t* (39) = −1.05, *p* = 0.298	0.028

**Notes.**

a Tested by paired sample *t* test.

b Measured by the Rosenberg’s Self-Esteem Scale (SES), with the score range being 10 and 40 and higher scores indicating better self-esteem.

c Measured by the Life Satisfaction Index A (LSIA), with the score range being 0 and 20 and higher scores indicating better life satisfaction.

d Measured by the Self-Rating Anxiety Scale (SAS), with the score range being 25 and 100 and higher scores indicating more severe anxiety symptoms.

e Measured by the Self-Rating Depression Scale (SDS), with the score range being 25 and 100 and higher scores indicating more severe depressive symptoms.

### Results with replacement for missing values

The results were mostly consistent with those from the original data set after replacing the missing values with series means except for the following. For consumers, the increase in self-esteem became significant (*t* (100) = −2.00, *p* = 0.048, ${\eta }_{p}^{2}$ = 0.039). For caregivers, the increase in life satisfaction became significant at 1 year (*t* (65) = −2.55, *p* = 0.013, ${\eta }_{p}^{2}$ = 0.091).

## Discussion

This study examined the effect of a 1-year community-based peer support program in two Chinese cities for persons suffering SMI. Overall, our findings highlighted that the community-based peer support services could be feasibly and sustainably developed in Chinese culture, with a measurable impact on participants’ well-being.

Peer support programs are beneficial in many places around the world (Repper & Carter, 2011; Suresh, Alam & Karkossa, 2021; Suresh et al., 2021). In the current study, we detected alleviation in psychiatric symptoms and improvement in personal and social functioning and life satisfaction after participation in the service among the Chinese population. It might be that peer support service offers a level of acceptance, understanding, and validation, which could be salutary to increase patients’ empowerment and hope and further facilitate their recovery (Suresh, Alam & Karkossa, 2021). Considering that the implementation of peer support services should be consistent with local customs, values, and resource availability (Solomon, 2004), the services were developed to conform to the Chinese culture and situation. For example, considering that the main source of support for people with mental illness in China is from their families (Lin, 1983), we also obtained approval from the family caregivers’ when recruiting consumers. In fact, we indeed observed that some consumers dropped out given their family members’ objections to the service. Peer service providers and community workers often need to work with family caregivers to ensure the participation of the consumers. Moreover, given that peer support service is relatively new in China, especially in communities. At the beginning of the project, both the caregivers and community workers were worried about the safety of the service, that is, what if the consumers would show acute psychiatric symptoms and hurt other consumers during the service sessions. Therefore, the recruited consumers were fairly stable in their psychiatric symptoms. In addition, the community workers would be immediately available when there are any emergencies during the services.

The study did not demonstrate a significant improvement in self-esteem among consumers, which was somewhat inconsistent with the outcomes of prior studies conducted in Western culture (Fukui et al., 2010; Lawn, Smith & Hunter, 2008; Mahlke et al., 2017). It might be that Chinese patients perceived more stigma of mental illness, given that patients with SMI are not widely accepted by the public, caregivers, and even the patients themselves. Patients are commonly reluctant to disclose their mental illness and it is fairly hard for them to be employed with a mental illness. In our study, the majority of the consumers were unemployed, which reflected the employment outcome for people with severe mental illness in urban China (Yang et al., 2013). Almost all of the patients (92.1%) received subsidies from the government. Although more than 60% of consumers and caregivers reported no or low financial issues, they might feel embarrassed given that they were depending on the government allowance and were unable to earn their own living. Therefore, it might be difficult to increase patients’ self-esteem in this context. However, after replacing the missing values with series means, the increase in consumers’ self-esteem became significant. This suggested that the current sample size might not be large enough to detect the increase in self-esteem among consumers. It might be that the improvement in self-esteem was more likely to be trivial and thus it needs a larger sample size to explore. Therefore, further research is needed to explore the impact of peer support and other community-based therapeutic programs on participants’ self-esteem or sense of self-efficacy, especially in the context of Chinese culture, with larger sample size.

Medication adherence did not increase significantly; however, this finding must be viewed in light of the relatively high level of adherence already present among our participants, including a substantial degree of self-administration. In our study, all of the consumers were expected to take prescribed medications regularly. Only a few participants reported medication non-adherence. This might be due to that the participants recruited in this study were fairly stable in their disease severity. Although one of the inclusion criteria of the consumers was mental illness conditions stable for at least 3 months, most of the recruited participants were stable in their mental conditions for several years. Considering that peer support service was developed at the community level of China for the first time and the peer service providers were lack of experience in dealing with emergencies, we included more stable patients to lower the probability of any critical situations, such as an episode of psychosis during the service sessions. Given that medication non-adherence is common among persons suffering SMI, future investigations with apparently less-cooperative participants will be important to examine the generalizability of peer programs such as this one. Self-selection bias could be an issue affecting clinically oriented studies such as this one (Fan, Shen & Tay, 2021).

Our previous qualitative research revealed that the majority of caregivers (96.9%) reported that ill family members participating in peer support service improved caregivers’ mood, increased their confidence in the recovery of ill family members, and reduced caretaker burdens (Fan et al., 2019). This result was not confirmed in the current study. These inconsistent findings might be due to that the quantitative measures used in the current study assessed the generic experience of self-esteem, life satisfaction, anxiety, and depressive symptoms, without identifying the specific content. It could be that the generic measures were not sufficient to capture the detailed aspects of caregivers’ life experiences, especially those caretaker burdens related aspects. Using different methods, our first study conducted interviews with caregivers, which allowed us to assess more specific benefits qualitatively, with a greater depth of inquiry and elaborated personal descriptions. This area of caregiver research among Chinese families requires further development, both quantitatively and qualitatively. Moreover, the sample size of caregivers is relatively small with a fairly high drop-out rate. Only the caregivers of those consumers who completed the follow-up evaluation were approached at 1 year in this study. After data imputation, the increase in caregivers’ life satisfaction became significant. This result is more consistent with our previous qualitative findings and other prior research (Fan et al., 2019; Lawn, Smith & Hunter, 2008), which implied that life satisfaction might be observed more evidently than other outcomes among caregivers. Further research may increase the sample size of caregivers and re-evaluate the changes in caregivers.

The findings of this study suggest several implications. First, this study revealed the outcomes of peer support service in Chinese culture, which enriched its implementation and effectiveness under diverse contexts and customs. Moreover, the intervention was implemented for a relatively long period and the participants were fairly stable. There were no adverse events or behavioral emergencies (*e.g.*, poorly controlled behavior, or problem that might require immediate attention) that occurred during the service sessions. The findings could support the sustainability of peer support services developed in Chinese culture and indicate the long-term effect of the service. Furthermore, not only consumers but also caregivers, who are also key stakeholders of peer support service, were followed up to evaluate the longitudinal effect of the service. Diverse outcomes were assessed, including not only the psychiatric symptoms and personal and social functioning evaluated by psychiatric professionals but also self-reported self-esteem and life satisfaction, which provided a more reliable and comprehensive understanding.

We recognize several important limitations to our work. We are aware that our design lent itself to the so-called “Hawthorne effect”. Rather resulting from the administration of specific active ingredients, the outcomes reflected the impact of nonspecific attention. To mitigate such effects, we would have needed a similarly composed control group, one with the same pretest and pos *t*-test assessments who received some type of attention. For example, control participants could have been invited to attend a comparable number of clinic sessions. Comparisons would have included the number of sessions attended and dropout rates, as well as before and after assessments.

A second concern involved attrition. Of the 101 initial participants, 23 dropped out. The reasons for the drop out at the 1-year follow-up include experiencing symptoms of relapse, not being supported by family members, and moving to a far place. However, we do not understand the specific factors associated with program discontinuation given that most of the dropouts were those who were only temporally unavailable during the follow-up assessment. They may come back and continue to participate in the service in the future. Therefore, more follow-up moments are needed to bring important information to the effect of this service and the factors that affect the program discontinuation. We also recognize that the measures used to assess outcomes of caregivers might be generic and insufficient to capture the specific experience of caretaker burdens. Future studies are encouraged to utilize more customized and detailed measures to evaluate the effect on caregivers. We also recognize that our study samples may have been skewed by participant bias; that is, persons with higher motivational levels chose to take part. This is another argument for a randomized trial with blinded outcome ratings. Additionally, we had no method to assure that our convenience samples recruited in Beijing and Chengdu were truly equivalent, despite measures on our scales, and we recognize that individuals found in these urban centers may not be representative of the broader national population of persons suffering SMI. Finally, we recognize that our participating community services were relatively well-resourced compared to many settings in China.

## Conclusions

Our findings indicate that peer support services could be effectively and sustainably implemented across China, with apparent positive impacts on participants suffering SMI. They suggest that community-based peer support services should be studied further, with the goal of defining their applicability to multiple, diverse urban and rural settings.

##  Supplemental Information

10.7717/peerj.14091/supp-1 File S1 Consumers raw data at baseline (T1) and follow-up (T2)

10.7717/peerj.14091/supp-2 File S2 Caregivers raw data at baseline (T1) and follow-up (T2)

10.7717/peerj.14091/supp-3 File S3 Consumer code for SPSS analysis

10.7717/peerj.14091/supp-4 File S4 Caregiver code for SPSS analysis

## Additional Information and Declarations

### Data Availability

The following information was supplied regarding data availability: The raw measurements are available in the Supplemental Files.
